# Quantitative Social Dialectology: Explaining Linguistic Variation Geographically and Socially

**DOI:** 10.1371/journal.pone.0023613

**Published:** 2011-09-01

**Authors:** Martijn Wieling, John Nerbonne, R. Harald Baayen

**Affiliations:** 1 Department of Humanities Computing, University of Groningen, Groningen, The Netherlands; 2 Department of Linguistics, University of Alberta, Edmonton, Canada; University of Maribor, Slovenia

## Abstract

In this study we examine linguistic variation and its dependence on both social and geographic factors. We follow dialectometry in applying a quantitative methodology and focusing on dialect distances, and social dialectology in the choice of factors we examine in building a model to predict word pronunciation distances from the standard Dutch language to 424 Dutch dialects. We combine linear mixed-effects regression modeling with generalized additive modeling to predict the pronunciation distance of 559 words. Although geographical position is the dominant predictor, several other factors emerged as significant. The model predicts a greater distance from the standard for smaller communities, for communities with a higher average age, for nouns (as contrasted with verbs and adjectives), for more frequent words, and for words with relatively many vowels. The impact of the demographic variables, however, varied from word to word. For a majority of words, larger, richer and younger communities are moving towards the standard. For a smaller minority of words, larger, richer and younger communities emerge as driving a change away from the standard. Similarly, the strength of the effects of word frequency and word category varied geographically. The peripheral areas of the Netherlands showed a greater distance from the standard for nouns (as opposed to verbs and adjectives) as well as for high-frequency words, compared to the more central areas. Our findings indicate that changes in pronunciation have been spreading (in particular for low-frequency words) from the Hollandic center of economic power to the peripheral areas of the country, meeting resistance that is stronger wherever, for well-documented historical reasons, the political influence of Holland was reduced. Our results are also consistent with the theory of lexical diffusion, in that distances from the Hollandic norm vary systematically and predictably on a word by word basis.

## Introduction

In this study we integrate the approaches of two fields addressing linguistic variation, dialectometry and (social) dialectology. Dialectology is the older discipline, where researchers focus on a single or small set of linguistic features in their analysis. Initially the focus in this field was on dialect geography [1, Ch. 2], where the distribution of these features was visualized on a map. Later, dialectologists more and more realized the importance of social variation. The work of Labov and later Trudgill has been very influential in this regard [2], [3]. Social dialectologists have often examined both social and linguistic influences on individual linguistic features, generally using logistic regression designs [4], but more recently also using mixed-effects regression modeling [5].

Dialectometry was pioneered by Jean Séguy, who calculated aggregate dialect distances based on the number of mismatching linguistic items between pairs of sites [6] and used a regression design to examine the influence of geography on these aggregate distances [7]. Since then other researchers, among others, Goebl, Heeringa and Nerbonne, and Kretzschmar, have refined the (computational and quantitative) techniques to measure and interpret these aggregate dialect distances [8]–[10]. We follow dialectometry in viewing linguistic distance for hundreds of individual words as our primary dependent variable.

2While the social dimension is a very important aspect in dialectology, it has been less important in dialectometry where the main focus still lies on dialect geography [11]. Of course there are some exceptions in which (for example) the diachronic perspective is taken into account [12], [13], or age and gender are considered as covariates [14], but to our knowledge no dialectometric study has attempted to model the effects of multiple geographic and social variables simultaneously.

Dialectometry has also been criticized for focusing too much on the aggregate level of linguistic differences [15], [16], thereby neglecting the level of linguistic structure where individual words and linguistic properties are important. Acknowledging honorable exceptions [11], we concede that the focus in dialectometry has been on aggregate levels, but the strength of the present analysis is that it focuses on individual words in addition to aggregate distances predicted by geography.

This quantitative social dialectological study is the first to investigate the effect of a range of social and lexical factors on a large set of dialect distances. In the following we will focus on building a model to explain the pronunciation distance between dialectal pronunciations (in different locations) and standard Dutch for a large set of distinct words. Of course, choosing standard Dutch as the reference pronunciation is not historically motivated, as standard Dutch is not the proto-language. However, the standard language remains an important reference point for two reasons. First, as noted by Kloeke, in the 16th and 17th centuries individual sound changes have spread from the Hollandic center of economic and political power to the more peripheral areas of the Netherlands [17]. Furthermore, modern Dutch dialects are known to be converging to the standard language [13], [18, pp. 355–356]. We therefore expect geographical distance to reveal a pattern consistent with Kloeke's ‘Hollandic Expansion’, with greater geographical distance correlating with greater distance from the Hollandic standard.

Kloeke also pointed out that sound changes may proceed on a word-by-word basis [17]. The case for lexical diffusion was championed by Wang and contrasts with the Neogrammarian view that sound changes are exceptionless and apply to all words of the appropriate form to undergo the change [19]. The Neogrammarian view is consistent with waves of sound changes emanating from Holland to the outer provinces, but it predicts that lexical properties such as a word's frequency of occurrence and its categorial status as a noun or verb should be irrelevant for predicting a region's pronunciation distance to the standard language.

In order to clarify the extent to which variation at the lexical level co-determines the dialect landscape in the Netherlands, we combine generalized additive modeling (which allows us to model complex non-linear surfaces) with mixed-effects regression models (which allow us to explore word-specific variation). First, however, we introduce the materials and methods of our study.

## Materials and Methods

### Pronunciation data

**Figure 1:**
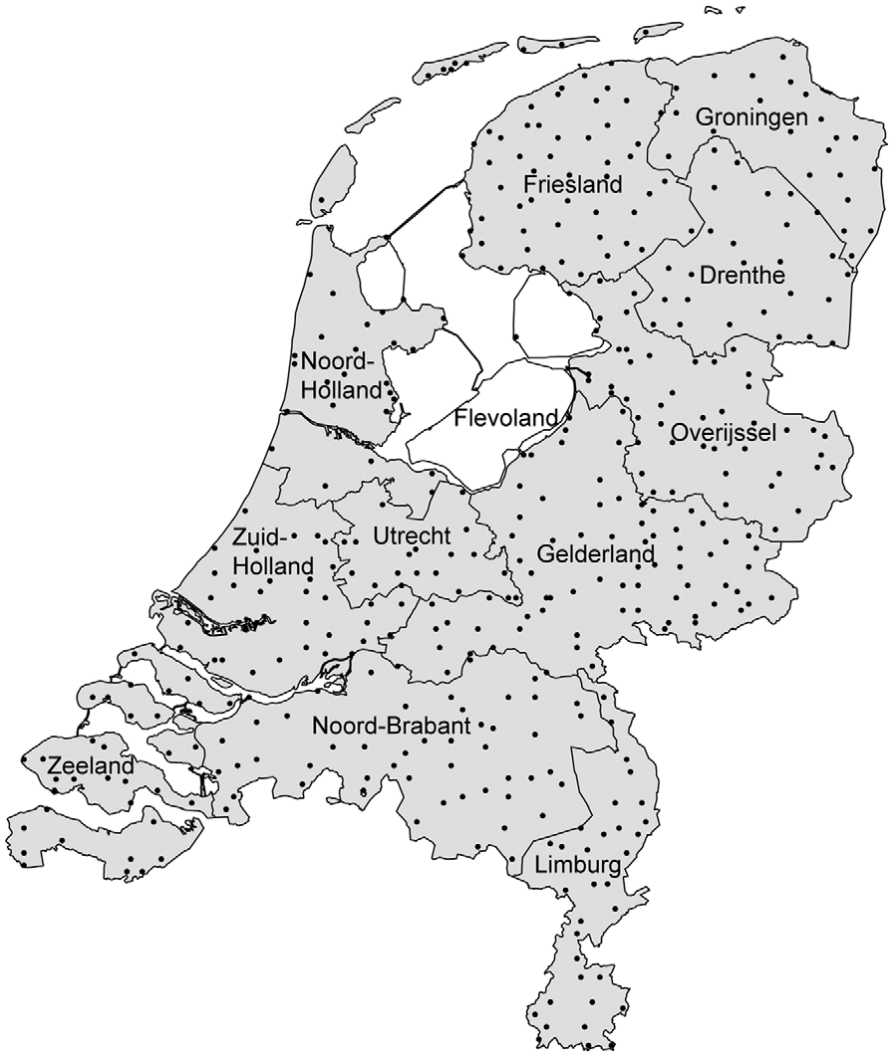
Distribution of locations in the GTRP including province names.

The Dutch dialect data set contains phonetic transcriptions of 562 words in 424 locations in the Netherlands. Figure 1 shows the distribution of the locations over the Netherlands together with the province names. Wieling, Heeringa and Nerbonne selected the words from the Goeman-Taeldeman-Van Reenen-Project (GTRP; [20]) specifically for an analysis of pronunciation variation in the Netherlands and Flanders [13]. The transcriptions in the GTRP were made by several transcribers between 1980 and 1995, making it currently the largest contemporary Dutch dialect data set available. The word categories include mainly verbs (30.8%), nouns (40.3%) and adjectives (20.8%). The complete list of words is presented in [13]. For the present study, we excluded 3 words of the original set (*gaarne*, *geraken* and *ledig*) as it turned out these words also varied lexically. The standard Dutch pronunciation of all 559 words was transcribed by one of the authors based on [21].

Because the set of words included common words (e.g., ‘walking’) as well as less frequent words (e.g., ‘oats’), we included word frequency information, extracted from the CELEX lexical database [22], as an independent variable.

### Social data

Besides the information about the speakers recorded by the GTRP compilers, such as year of recording, gender and age of the speaker, we extracted additional demographic information about each of the 424 places from Statistics Netherlands [23]. We obtained information about the average age, average income, number of inhabitants (i.e. population size) and male-female ratio in every location in the year 1995 (approximately coinciding with the end of the GTRP data collection period). As Statistics Netherlands uses three measurement levels (i.e. neighborhood, district and municipality), we manually selected the appropriate level for every location. For large cities (e.g., Rotterdam), the corresponding municipality (generally having the same name) was selected as it mainly consisted of the city itself. For smaller cities, located in a municipality having multiple villages and/or cities, the district was selected which consisted of the single city (e.g., Coevorden). Finally, for very small villages located in a district having multiple small villages, the neighborhood was selected which consisted of the single village (e.g., Barger-Oosterveld).

### Obtaining pronunciation distances

For all 424 locations, the pronunciation distance between standard Dutch and the dialectal pronunciations was calculated by using the Levenshtein distance [24]. The Levenshtein distance minimizes the number of insertions, deletions and substitutions to transform one pronunciation string into the other. For example, the Levenshtein distance between two Dutch dialectal variants of the word ‘two’, [tei] and [twa], is 3:
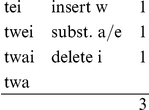
The corresponding alignment is:
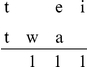
Note that in the example above an alternative optimal alignment substitutes [a] for [i] instead of [e].

The regular Levenshtein distance does not distinguish vowels and consonants and therefore may align a vowel with a consonant. To enforce linguistically sensible alignments, a syllabicity constraint is normally added such that vowels are not aligned with (non-sonorant) consonants.

As shown in the example above, the Levenshtein distance increases with one for every mismatch. Some sounds, however, are phonetically closer to each other than other sounds, e.g., /a/ and /e/ are closer than /a/ and /i/. A distance measure for two pronunciations should reflect this. Wieling, Prokić and Nerbonne introduced a method which uses the relative alignment frequency of sounds to determine their distance [25]. Pairs of sounds which are aligned relatively frequently are assigned a low distance, while sounds which co-occur relatively infrequently are assigned a high distance. The method is based on calculating the Pointwise Mutual Information score (PMI; [26]) between every pair of sounds and was found to improve alignments compared to the Levenshtein distance with (and without) the syllabicity constraint. In addition, a recent study by Wieling, Margaretha and Nerbonne (submitted) found that the automatically determined PMI distances between vowels correspond well with acoustic vowel distances for several languages. A detailed description about the PMI method can be found in [27].

As an illustration of the PMI method, consider the alignment of [tei] and [twa], now using the PMI-based costs:
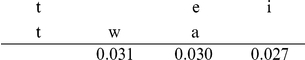
In contrast to the previous example, the [a] can only be aligned with [e], as the cost of aligning [a] and [i] is higher (and the cost of deleting [e] is higher than deleting [i]).

In the following, the pronunciation distances are based on the PMI-based Levenshtein distance. Because longer words will likely have a greater pronunciation distance (as more sounds may change) than shorter words, we normalize the PMI-based word pronunciation distances by dividing by the alignment length.

### Modeling the role of geography: generalized additive modeling

Given a fine-grained measure capturing the distance between two pronunciations, a key question from a dialectometric perspective is how to model pronunciation distance as a function of the longitude and latitude of the pronunciation variants. The problem is that for understanding how longitude and latitude predict pronunciation distance, the standard linear regression model is not flexible enough. The problem with standard regression is that it can model pronunciation distance as a flat plane spanned by longitude and latitude (by means of two simple main effects) or as a hyperbolic plane (by means of a multiplicative interaction of longitude by latitude). A hyperbolic plane, unfortunately, imposes a very limited functional form on the regression surface that for dialect data will often be totally inappropriate.

We therefore turned to generalized additive models (GAM), an extension of multiple regression that provides flexible tools for modeling complex interactions describing wiggly surfaces. For isometric predictors such as longitude and latitude, thin plate regression splines are an excellent choice. Thin plate regression splines model a complex, wiggly surface as a weighted sum of geometrically simpler, analytically well defined, surfaces [28]. The details of the weights and smoothing basis functions are not of interest for the user, they are estimated by the GAM algorithms such that an optimal balance between undersmoothing and oversmoothing is obtained, using either generalized cross-validation or relativized maximum likelihood (see [29] for a detailed discussion). The significance of a thin plate regression spline is assessed with an *F*-test evaluating whether the estimated degrees of freedom invested in the spline yield an improved fit of the model to the data. Generalized additive models have been used successfully in modeling experimental data in psycholinguistics, see [30] for evoked response potentials, and see [31]–[33] for chronometric data. They are also widely used in biology, see, for instance, [34] for spatial explicit modeling in ecology.

**Figure 2:**
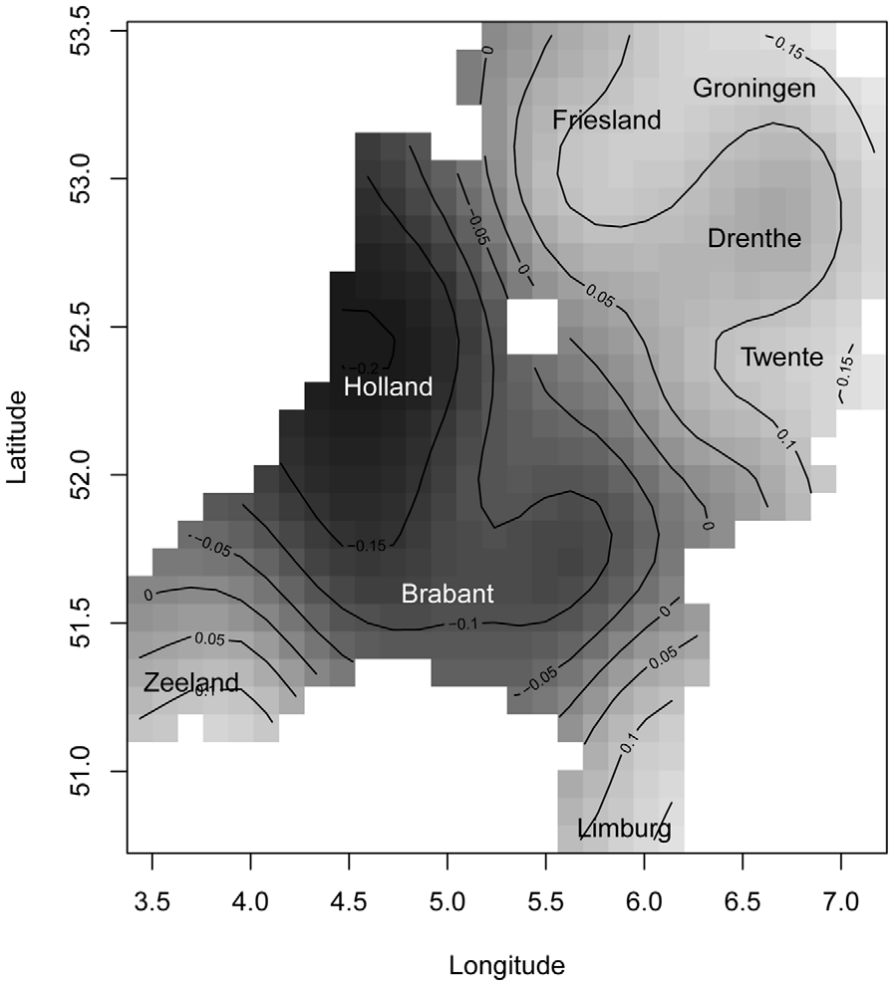
Contour plot obtained with a generalized additive model. The contour plot shows a regression surface of pronunciation distance as a function of longitude and latitude obtained with a generalized additive model using a thin plate regression spline. The (black) contour lines represent distance isoglosses, darker shades of gray indicate smaller distances closer to the standard language, lighter shades of gray represent greater distances. Note that the empty square indicates the location of the IJsselmeer, a large lake in the Netherlands.

For our data, we use a generalized additive model to provide us with a two-dimensional surface estimator (based on the combination of longitude and latitude) of pronunciation distance using thin-plate regression splines as implemented in the *mgcv* package for *R* [29]. Figure 2 presents the resulting regression surface using a contour plot. The (solid) contour lines represent distance isoglosses. Darker shades of gray indicate smaller distances, lighter shades of gray represent greater distances from the standard language.

The general geographic pattern fits well with Kloeke's hypothesis of a Hollandic expansion: As we move away from Holland, pronunciation distances increase [17]. Kloeke showed that even in the sixteenth and seventeenth centuries the economic and political supremacy of the provinces of North and South Holland led to the spread of Hollandic speech norms to the outer provinces.

We can clearly identify the separation from the standard spoken in the provinces of North and South Holland (central west) of the province of Friesland (in the north), the Low Saxon dialects spoken in Groningen and Drenthe (in the northeast), and the Franconian dialects of Zeeland (in the southwest) and Limburg (southeast). The 28.69 estimated degrees of freedom invested in the thin plate regression spline were supported by an *F*-value of 1051 (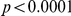). The local cohesion in Figure 2 makes sense, since nearby locations tend to speak dialectal varieties which are relatively similar [35].

### Mixed-effects modeling

A problem with this generalized additive model is that the random-effects structure of our data set is not taken into account. In mixed-effects regression modeling (for introductions, see, e.g., [36]–[38]), a distinction is made between fixed-effect and random-effect factors. Fixed-effect factors are factors with a small number of levels that exhaust all possible levels (e.g., the gender of a speaker is either male or female). Random-effect factors, by contrast, have levels sampled from a much larger population of possible levels. In our data, there are three random-effect factors that are likely to introduce systematic variation that is ignored in our generalized additive model.

A first random-effect factor is location. Our observations are made at 424 locations where speakers were interviewed. Since these 424 locations are a sample of a much larger set of communities that might have been sampled, location is a random-effect factor. Because we used the pronunciations of a single speaker at a given location, location is confounded with speaker. Hence, our random-effect factor location represents both location and speaker.

The data obtained from the 424 locations were coded phonetically by 30 different transcribers. Since these transcribers are themselves a sample of a larger set of possible transcribers, transcriber is a second random-effect factor in our model. By including transcriber in our model, we can account for biases in how individuals positioned the data that they listened to with respect to the standard language.

The third random-effect factor is word. Each of the 559 words was pronounced in most of the 424 locations. The words are also sampled from a much larger population of words, and hence constitute a random-effect factor as well.

In mixed-effect models, random-effect factors are viewed as sources of random noise that can be linked to specific observational units, in our case, locations, transcribers, and words. In the simplest case, the variability associated with a given random-effect factor is restricted to adjustments to the population intercept. For instance, some transcribers might be biased towards the standard language, others might be biased against it. These biases are assumed to follow a normal distribution with mean zero and unknown standard deviation to be estimated from the data. Once these biases have been estimated, it is possible to adjust the population intercept so that it becomes precise for each individual transcriber. We will refer to these adjusted intercepts as – in this case – by-transcriber random intercepts.

It is possible, however, that the variation associated with a random-effect factor affects not only the intercept, but also the slopes of other predictors. We shall see below that in our data the slope of population size varies with word, indicating that the strength of population size is not the same for all words. A mixed-effects model will estimate the by-word biases in the slope of population size, and by adding these estimated biases to the general population size slope, by-word random slopes are obtained that make the estimated effect of population size as precise as possible for each word.

Whether random intercepts and random slopes are justified is verified by means of likelihood ratio tests, which evaluate whether the increase in the number of parameters is justified given the increase in goodness of fit.

Statistical models combining mixed-effects regression and generalized additive modeling are currently under development. We have explored the *gamm4* package for *R* developed by Wood, but this package proved unable to cope with the rich random effects structure characterizing our data. We therefore used the generalized additive model simply to predict the pronunciation distance from longitude and latitude, without including any further predictors. We then use the fitted values of this simple model (see Figure 2) as a predictor representing geography in our final model. (The same approach was taken by Schmidt and colleagues, who also failed to use the *gamm4* package successfully [34].) In what follows, we refer to these fitted values as the GAM distance.

In our analyses, we considered several other predictors in addition to GAM distance and the three random-effect factors location, transcriber, and word. We included a contrast to distinguish nouns (and adverbs, but those only occur infrequently) from verbs and adjectives. Other lexical variables we included were word frequency, the length of the word, and the vowel-to-consonant ratio in the standard Dutch pronunciation of each word. The location-related variables we investigated were average age, average income, male-female ratio and the total number of inhabitants in every location. Finally, the speaker- and transcriber-related variables we extracted from the GTRP were gender, year of birth, year of recording and gender of the fieldworker (not necessarily being the same person as the transcriber). Unfortunately, for about 5% of the locations the information about gender, year of birth and year of recording was missing. As information about the employment of the speaker or speaker's partner was missing even more frequently (in about 17% of the locations), we did not include this variable in our analysis.

A recurrent problem in large-scale regression studies is collinearity of the predictors. For instance, in the Netherlands, communities with a larger population and higher average income are found in the west of the country. In order to facilitate interpretation, and to avoid enhancement or suppression due to correlations between the predictor variables [39], we decorrelated such predictors from GAM distance by using as predictor the residuals of a linear model regressing that predictor on GAM distance. For average age as well as for population count, the resulting residuals correlated highly with the original values (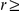
0.97), indicating that the residuals can be interpreted in the same way as the original values. Because average income and average population age were also correlated (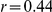) we corrected the variable representing the average population age for the effect of average income.

In order to reduce the potentially harmful effect of outliers, various numerical predictors were log-transformed. We scaled all numerical predictors by subtracting the mean and dividing by the standard deviation in order to facilitate the interpretation of the fitted parameters of the statistical model. Our dependent variable, the pronunciation distance per word from standard Dutch (averaged by alignment length) was also log-transformed and centered. The value 0 indicates the mean distance from the standard pronunciation, while negative values indicate a distance closer and positive values indicate a distance farther away from standard Dutch.

The significance of fixed-effect predictors was evaluated by means of the usual *t*-test for the coefficients, in addition to model comparison likelihood ratio tests and AIC (Akaike Information Criterion; [40]). Since our data set contains a very large number of observations (a few hundred thousand items), the *t*-distribution approximates the standard normal distribution and factors will be significant (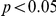) when they have an absolute value of the *t*-statistic exceeding 2 [37]. A one-tailed test (only applicable with a clear directional hypothesis) is significant when the absolute value of the *t*-statistic exceeds 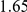.

## Results

The total number of cases in our original data set was 228,476 (not all locations have pronunciations for every word). To reduce the effect of noise in the transcriptions, we eliminated all items in our data set with a pronunciation distance from standard Dutch larger than 2.5 standard deviations above the mean pronunciation distance for each word. Because locations in the province of Friesland are characterized by having a separate language (Frisian) with a relatively large distance from standard Dutch, we based the exclusion of items on the means and standard deviation for the Frisian and non-Frisian area separately. After deleting 2610 cases (1.14%), our final data set consisted of 225,866 cases.

We fitted a mixed-effects regression model to the data, step by step removing predictors that did not contribute significantly to the model fit. In the following we will discuss the specification of the resulting model including all significant predictors and verified random-effect factors. This model explains approximately 44.5% of the variance of our dependent variable (i.e. the linguistic distance compared to standard Dutch).

The coefficients and associated statistics of the fixed-effect factors and covariates are shown in Table 1 (note that most values in the table are close to 0 as we are predicting average PMI distances, which are relatively small). The random-effect structure is summarized in Table 2. The residuals of our model followed a normal distribution, and did not reveal any non-uniformity with respect to location. Table 3 summarizes the relation between the independent variables and the distance from standard Dutch. A more detailed interpretation is provided in the sections below on demographic and lexical predictors.

**Table 1:** Fixed-effect coefficients of a minimally adequate model fitted to the pronunciation distances from standard Dutch.

	Estimate	Std. Error	*t*-value
Intercept	−0.0153	0.0105	−1.4561
GAM distance (geography)	0.9684	0.0274	35.3239
Population size (log)	−0.0069	0.0026	−2.6386
Population average age	0.0045	0.0025	1.8049
Population average income (log)	−0.0005	0.0026	−0.1988
Word frequency (log)	0.0198	0.0060	3.2838
Noun instead of Verb/Adjective	0.0409	0.0122	3.3437
Vowel-consonant ratio (log)	0.0625	0.0059	10.5415

**Table 2:** Random-effect parameters of the minimally adequate model fitted to the pronunciation distances from standard Dutch.

Factors	Rnd. effects	Std. Dev.	Cor.	
Word	Intercept	0.1394		
	Pop. size (log)	0.0186		
	Pop. avg. age	0.0086	−0.856	
	Pop. avg. income (log)	0.0161	0.867	−0.749
Location	Intercept	0.0613		
	Word freq. (log)	0.0161	−0.084	
	Noun instead of Verb/Adj.	0.0528	−0.595	0.550
Transcriber	Intercept	0.026		
Residual		0.2233		

The column Cor. contains the correlations between the random slopes and/or intercepts. The first number in the first correlation column for the by-word random slopes represents the correlation between the by-word random slope for population size and the by-word random slope for average age, while the second number represents the correlation between the by-word random slope for population size and the by-word random slope for average income. The first number in the second column represents the correlation between the by-word random slopes of average income and average age. Similarly, the first correlation column for the by-location random slopes contains the correlations between the by-location random intercept and the random slope for word frequency and the noun-verb contrast, respectively. The second column contains the correlation between the by-location random slopes for word frequency and the noun-verb contrast. See the text for interpretation.

**Table 3:** Interpretation of significant fixed-effect predictors.

Predictor	Interpretation
GAM distance (geography)	Peripheral areas in the north, east and south have a higher distance from standard Dutch than the central western part of the Netherlands (see Figure 2).
Population size (log)	Locations with a larger population have a pronunciation closer to standard Dutch, but the effect varies per word (see Figure 3).
Population average age	Locations with a younger population have a pronunciation closer to standard Dutch, but the effect varies per word (see Figure 3).
Population average income (log)	There is no significant general effect of average income in a population, but the effect varies per word (see Figure 3).
Word frequency (log)	More frequent words have a higher distance from standard Dutch, but the effect varies per location (see Figure 4).
Noun instead of Verb/Adjective	Nouns have a higher distance from standard Dutch than verbs and adjectives, but the effect varies per location (see Figure 5).
Vowel-consonant ratio (log)	Words with relatively more vowels have a higher distance from standard Dutch.

The inclusion of the fixed-effect factors (except average population income) and random-effect factors shown in Table 1 and 2 was supported by likelihood ratio tests indicating that the additional parameters significantly improved the goodness of fit of the model. Tables 4 and 5 show the increase of the goodness of fit for every additional factor measured by the increase of the log-likelihood and the decrease of the Akaike Information Criterion [40]. To assess the influence of each additional fixed-effect factor, the random effects were held constant, including only the random intercepts for word, location and transcriber. The baseline model, to which the inclusion of the first fixed-effect factor (geography) was compared, only consisted of the random intercepts for word, location and transcriber. Subsequently, the next model (including both geography and the vowel-to-consonant ratio per word), was compared to the model including geography (and the random intercepts) only. This is shown in Table 4 (sorted by decreasing importance of the individual fixed-effect factors). Log-likelihood ratio tests were carried out with maximum likelihood estimation, as recommended in [36].

**Table 4:** Goodness of fit of the fixed-effect factors of the model.

	Log-lik. increase	AIC decrease	Likelihood ratio test
Random intercepts			
+GAM distance (geography)	270.6	539.2	p 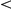 0.0001
+Vowel-consonant ratio (log)	50.9	99.8	p 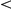 0.0001
+Noun instead of Verb/Adjective	5.6	9.2	p 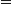 0.0008
+Population size	3.8	5.7	p 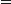 0.0056
+Word frequency (log)	3.8	5.7	p 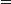 0.0056
+Population average age	2.5	3.1	p 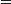 0.0244
+Population average income (log)	0.0	−2.0	p 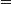 0.9554

Each row specifies the increase in goodness of fit obtained by adding the current predictor to the model including all preceding predictors (as well as the random intercepts for word, location and transcriber). Note that the final row indicates that population average income does not improve the model.

**Table 5:** Goodness of fit of the random-effect factors of the model.

	Log-lik. increase	AIC decrease	Likelihood ratio test
Fixed-effect factors			
+Random intercept word	32797.8	65593.6	p 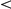 0.0001
+Random intercept location	5394.2	10786.4	p 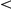 0.0001
+Random intercept transcriber	14.0	26.1	p 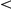 0.0001
+Population size (word)	490.3	978.6	p 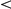 0.0001
+Population average age (word)	96.0	188.0	p 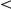 0.0001
+Population average income (word)	443.9	881.8	p 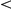 0.0001
+Word frequency (location)	220.1	436.3	p 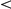 0.0001
+Noun instead of Verb/Adj. (location)	1064.4	2122.8	p 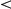 0.0001

Each row specifies the increase in goodness of fit of the model resulting from the addition of the specified random slope or intercept to the preceding model. All models include the fixed effect factors listed in Table 1.

Similarly, the importance of additional random-effect factors was assessed by restricting the fixed-effect predictors to those listed in Table 1. The baseline model in Table 5, to which the inclusion of the random intercept for word was compared, only consisted of the fixed-effect factors listed in Table 1. The next model (also including location as a random intercept) was compared to the model with only word as a random intercept. In later steps random slopes were added. For instance, the sixth model (including random slopes for population size and average population age, and their correlation) was compared to the fifth model which only included population size as a random slope. Log-likelihood ratio tests evaluating random-effects parameters were carried out with relativized maximum likelihood estimation, again following [36].

Due to the large size of our data set, it proved to be computationally infeasible to include all variables in our random-effects structure (e.g., the vowel-to-consonant ratio was not included). As further gains in goodness of fit are to be expected when more parameters are invested in the random-effects structure, our model does not show the complete (best) random-effects structure. However, we have checked that the fixed-effect factors remained significant when additional uncorrelated by-location or by-word random slopes were included in the model specification. In other words, we have verified that the *t*-values of the fixed-effect factors in Table 1 are not anti-conservative and therefore our results remain valid.

### Demographic predictors

The geographical predictor GAM distance (see Figure 2) emerged as the predictor with the smallest uncertainty concerning its slope, as indicated by the huge *t*-value. As GAM distance represents the fitted values of a generalized additive model fitted to pronunciation distance from standard Dutch (adjusted 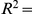
0.12), the strong statistical support for this predictor is unsurprising. Even though GAM distance accounts for a substantial amount of variance, location is also supported as a significant random-effect factor, indicating that there are differences in pronunciation distances from the standard language that cannot be reduced to geographical location. The random-effect factor location, in other words, represents systematic variability that can be traced to the different locations (or speakers), but that resists explanation through our demographic fixed-effect predictors. To what extent, then, do these demographic predictors help explain pronunciation distance from the standard language over and above longitude, latitude, and the location (speaker) itself?

Table 1 lists two demographic predictors that reached significance. First, locations with many inhabitants (a large population size) tend to have a lower distance from the standard language than locations with few inhabitants. A possible explanation for this finding is that people tend to have weaker social ties in urban populations, which causes dialect leveling [41]. Since the standard Dutch language has an important position in the Netherlands [18], [42], and has been dominant for many centuries [17], conversations between speakers of different dialects will normally be held in standard Dutch and consequently leveling will proceed in the direction of standard Dutch. The greater similarity of varieties in settlements of larger size is also consistent with the predictions of the gravity hypothesis which states that linguistic innovation proceeds first from large settlements to other large nearby settlements, after which smaller settlements adopt the innovations from nearby larger settlements [43].

The second (one-tailed) significant demographic covariate is the average age of the inhabitants of a given location. Since younger people tend to speak less in their dialect and more in standard Dutch than the older population [12], [18, pp. 355–356], the positive slope of average age is as expected.

Note that Table 1 also contains average income as a demographic covariate. This variable is not significant in the fixed-effect part of the model (as the absolute *t*-value is lower than 1.65), but is included as it is an important predictor in the random-effects structure of the model.

**Figure 3:**
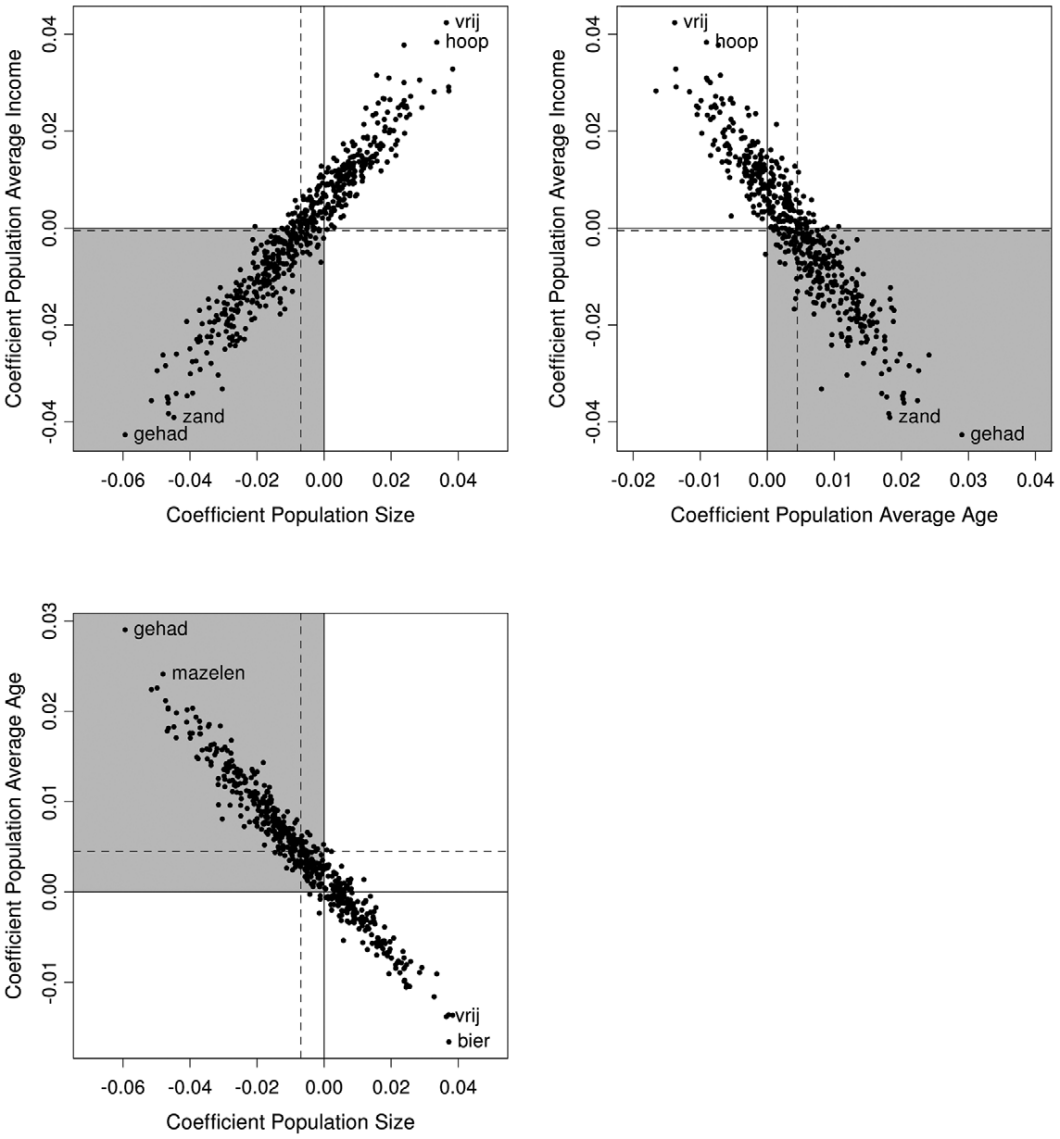
By-word random slopes in a mixed-effects model fitted to pronunciation distances from standard Dutch. All combinations of by-word random slopes (i.e. the word-specific coefficients) for population size, age and income are shown. The grey quadrant in every graph marks where most words (dots) are located. The dashed lines indicate the model estimates of every predictor.

Interestingly, all three demographic predictors required by-word random slopes. Figure 3 shows the by-word random slopes for all combinations of population size (i.e. the number of inhabitants), average age and average income. At the extremes in every graph, the words themselves have been added to the scatter plot (*gehad*, ‘had’; *zand*, ‘sand’; *hoop*, ‘hope’; *vrij*, ‘free’; *mazelen*, ‘measles’; *bier*, ‘beer’). The grey quadrant in every graph marks where most words are located. Words in this quadrant have slopes consistent with the general model (the model estimates shown in Table 1 are indicated by the dashed lines).

When looking at the top-left graph, we see that most words (represented by dots) are located in the lower left quadrant, consistent with the negative slope of population size (−0.0069) and the (non-significant) negative slope of average income (−0.0005; see Table 1). Words in this quadrant have negative slopes for population size, indicating that these words will tend to be more similar to the standard in larger communities (the more to the left the dot is located, the more similar it will be to the standard language). At the same time, the same words also have negative slopes for average income, indicating that these words will tend to be more similar to the standard in richer communities (the lower the dot is located, the more similar it will be to the standard language). This pattern reverses for the words in the opposite quadrant. A word such as *vrij* (free) has a large positive coefficient for population size, indicating that in larger communities this word will differ more from the standard. The word *vrij* also has a positive coefficient for average income. Therefore, speakers in poorer communities will pronounce the word closer to the standard, while speakers in richer communities will pronounce it more differently. The correlation parameter of 0.867 in Table 2 quantifies the strong connection between the by-word random slopes for average income and population size.

The top-right graph illustrates that the coefficients of average age and average income are also closely linked per word (indicated by the high correlation parameter of −0.749 in Table 2). Words in the grey quadrant behave in accordance with the general model (e.g., the word *gehad* will be more similar to the standard language in a richer community as well as in a younger community), while words in the opposite quadrant behave in a reversed fashion (e.g., the word *vrij* will differ more from the standard in a richer community as well as in a younger community).

Finally, the bottom-left graph shows that the coefficients of population size and average age are also closely connected per word (indicated by the high correlation parameter of −0.856 in Table 2). Words in the grey quadrant behave in accordance with the general model (e.g., the word *gehad* will be more similar to the standard language in a larger community as well as in a younger community), while words in the opposite quadrant behave in a reversed fashion (e.g., the word *bier* will differ more from the standard in a larger community as well as in a younger community).

Two important points emerge from this analysis. First, the effects of the three demographic variables, population size, average age and average income, differ dramatically depending on what word is being considered. Second, words tend to be influenced by all three demographic variables similarly. If a word is influenced more strongly by one variable than predicted by the general model, it will also be influenced more strongly by the other two variables (e.g., the word *gehad*). Alternatively, if a word is influenced in the reverse direction by one variable compared to the general model, it will likely also be influenced in the reverse direction by the other two variables (e.g., the word *vrij*).

Besides these significant variables, we investigated several other demographic predictors that did not reach significance. One variable we considered was the male-female ratio at a given location. While the gender of the speaker is likely to play an important role, we are uncertain if the ratio of men versus women in a location should play a significant role. With other predictors in the model, it did not prove significant. We also expected a negative influence of average income on the pronunciation distance from the standard, since standard Dutch has a relatively high prestige [18, Ch. 12]. However, as shown in Table 1, this effect did not reach significance, possibly due to the large collinearity with geography; the highest average income in the Netherlands is earned in the western part of the Netherlands [23], where dialects are also most similar to standard Dutch [44, p. 274]. Average income was highly significant when geography was excluded from the model.

No speaker-related variables were included in the final model. We were surprised that the gender of the speaker did not reach significance, as the importance of this factor has been reported in many sociolinguistic studies [45]. However, when women have a limited social circle (e.g., the wife of a farmer living on the outskirts of a small rural community), they actually tend to speak more traditionally than men [18, p. 365]. Since such women are certainly present in our data set, this may explain the absence of a gender difference in our model. We also expected speaker age to be a significant predictor, since dialects are leveling in the Netherlands [12], [18, pp. 355–356]. However, as the speakers were relatively close in age (e.g., 74% of the speakers were born between 1910 and 1930) and we only used pronunciations of a single speaker per location, this effect might have been too difficult to detect in our data set.

The two fieldworker-related factors (gender of the fieldworker and year of recording) were not very informative, because they suffered from substantial geographic collinearity. With respect to the year of recording, we found that locations in Friesland were visited quite late in the project, while their distances from standard Dutch were largest. Regarding the gender of the fieldworkers, female fieldworkers mainly visited the central locations in the Netherlands, while the male fieldworkers visited the more peripheral areas (where the pronunciation distance from standard Dutch is larger).

### Lexical predictors

Table 1 lists three lexical predictors that reached significance: the vowel-to-consonant ratio, word frequency and the contrast between nouns and verbs. Unsurprisingly, the length of the word was not a significant predictor, as we normalized pronunciation distance by the alignment length.

The first significant lexical factor was the vowel-to-consonant ratio. The general effect of the vowel-to-consonant ratio was linear, with a greater ratio predicting a greater distance from the standard. As vowels are much more variable than consonants (e.g., [46]), this is not a very surprising finding.

The second, more interesting, significant lexical factor was word frequency. More frequent words tend to have a higher distance from the standard. We remarked earlier that Dutch dialects tend to converge to standard Dutch. A larger distance from the standard likely indicates an increased resistance to standardization. Indeed, given the recent study of Pagel and colleagues, where they show that more frequent words are more resistant to change [47], this seems quite sensible.

**Figure 4:**
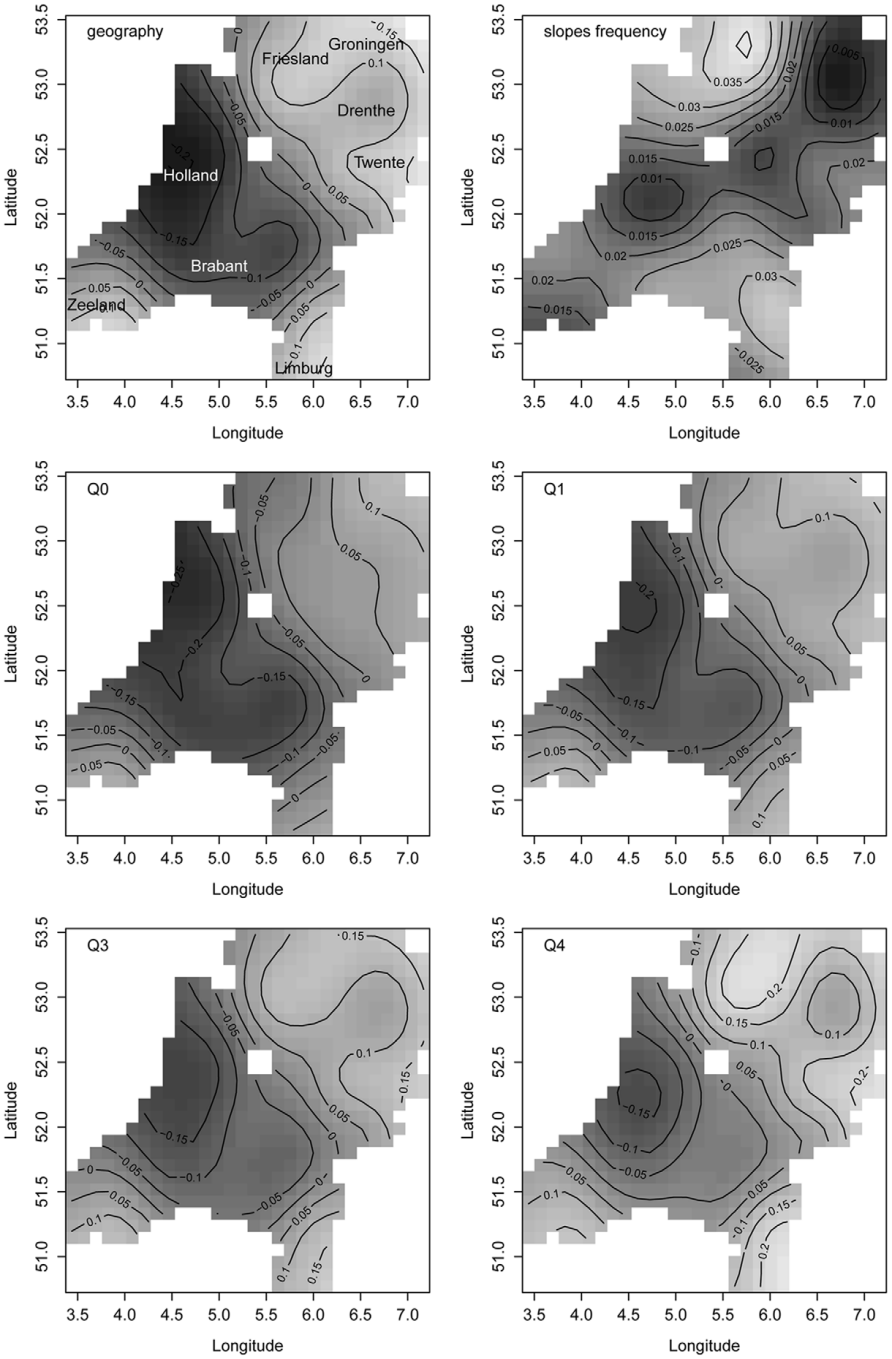
Word frequency and distance from the standard language. Upper left: distance predicted only from longitude and latitude. Upper right: the geographical distribution of random slopes for word frequency. Lower four panels: the combined effect of geography and word frequency on pronunciation distance for the minimum frequency (Q0), the first (Q1) and third quartile (Q3) and the maximum frequency (Q4). Darker shades of gray denote smaller values, lighter shades indicate larger values.

However, the effect of word frequency is not uniform across locations, as indicated by the presence of by-location random slopes for word frequency in our model (see Table 2). The parameters for these random slopes (the standard deviation for the random slopes and the correlation parameter for the random slopes and intercepts) jointly increase the log-likelihood of the model by no less than 220 units, compared to 3.8 log-likelihood units for the fixed-effect (population) slope of frequency. Interestingly, although the by-location random slopes for frequency properly follow a normal distribution, they are not uniformly distributed across the different regions of the Netherlands, as illustrated in the upper right panel of Figure 4. In this panel, contour lines link locations for which the slope of the frequency effect is the same. The two dark grey areas (central Holland and Groningen and Drenthe) are characterized by slopes close to zero, while the white area in Friesland indicates a large positive slope (i.e. the Frisian pronunciations become more distinct from standard Dutch for higher frequency words).

To clarify how geography (GAM distance) and frequency jointly predict distance from the standard language, we first calculated the fitted GAM distance for each location. We then estimated the predicted distance from the standard language using GAM distance and word frequency as predictors, weighted by the weights estimated by our mixed-effects model. Because the fitted surfaces vary with frequency, we selected the minimum frequency (Q0), first (Q1) and third (Q3) quartiles as well as the maximum frequency (Q4) for visualization (see the lower panels in Figure 4). Panel Q0 shows the surface for the words with the lowest frequency in our data. As frequency increased, the surface gradually morphs into the surface shown in the lower right panel (Q4). The first thing to note is that as frequency increases, the shades of grey become lighter, indicating greater differences from the standard. This is the main effect of frequency: higher-frequency words are more likely to resist assimilation to the standard language. The second thing to note is that the distances between the contour lines decrease with increasing frequency, indicating that the differences between regions with respect to the frequency effect become increasingly more pronounced. For instance, the Low Saxon dialect of Twente on the central east border with Germany, and the Frisian varieties in the north profile themselves more clearly as different from the Hollandic standard for the higher-frequency words (Q4) than for the lower-frequency words (Q0).

For the lowest-frequency words (panel Q0), the northeast separates itself from the Hollandic sphere of influence, with distance slowly increasing towards the very northeast of the country. This area includes Friesland and the Low Saxon dialects. As word frequency increases, the distance from standard Dutch increases, and most clearly so in Friesland. For Friesland, this solid resistance to the Hollandic norm, especially for high-frequency words, can be attributed to Frisian being a different language that is mutually unintelligible with standard Dutch.

Twente also stands out as highly resistant to the influence of the standard language. In the 16th and 17th centuries, this region was not under firm control of the Dutch Republic, and Roman Catholicism remained stronger here than in the regions towards its west and north. The resistance to protestantism in this region may have contributed to its resistance to the Hollandic speech norms (see also [48]).

In the southwest (Zeeland) and the southeast (Limburg), we find Low Franconian dialects that show the same pattern across all frequency quartiles, again with increased distance from Holland predicting greater pronunciation distance. The province of Limburg has never been under firm control of Holland for long, and has a checkered history of being ruled by Spain, France, Prussia, and Austria before becoming part of the kingdom of the Netherlands. Outside of the Hollandic sphere of influence, it has remained closer to dialects found in Germany and Belgium. The province of Zeeland, in contrast, has retained many features of an earlier linguistic expansion from Flanders – in the middle ages, Flanders had strong political influence in Zeeland. Zeeland was not affected by an expansion from Brabant (which is found in the central south of the Netherlands as well as in Belgium), but that expansion strongly influenced the dialects of Holland. This Brabantic expansion, which took place in the late middle ages up to the seventeenth century, clarifies why, across all frequency quartiles, the Brabantic dialects are most similar to the Hollandic dialects.

Our regression model appears to conflict with the view of Kloeke (which was also adopted by Bloomfield) that high-frequency words should be more likely to undergo change than low-frequency words [17], [49]. This position was already argued for by Schuchardt, who discussed data suggesting that high-frequency words are more profoundly affected by sound change than low-frequency words [50]. Bybee called attention to language-internal factors of change that are frequency-sensitive [51]. She argued that changes affecting high-frequency words first would be a consequence of the overlap and reduction of articulatory gestures that comes with fluency. In contrast, low-frequency words would be more likely to undergo analogical leveling or regularization.

Our method does not allow us to distinguish between processes of articulatory simplification and processes of leveling or regularization. Moreover, our method evaluates the joint effect of many different sound changes for the geographical landscape. Our results indicate that, in general, high-frequency words are most different from the standard. However, high-frequency words can differ from the standard for very different reasons. For instance, they may represent older forms that have resisted changes that affected the standard. Alternatively, they may have undergone region-specific articulatory simplification. Furthermore, since higher-frequency forms are better entrenched in memory [52], [53], they may be less susceptible to change. As a consequence, changes towards the standard in high-frequency words may be more salient, and more likely to negatively affect a speaker's in-group status as a member of a dialect community. Whatever the precise causes underlying their resistance to accommodation to the standard may be, our data do show that the net outcome of the different forces involved in sound change is one in which it is the high-frequency words that are most different from the standard language.

The third lexical factor that reached significance was the contrast between nouns as opposed to verbs and adjectives. Nouns have a greater distance from the standard language than verbs and adjectives. (Further analyses revealed that the effects of verbs and adjectives did not differ significantly.) This finding harmonizes well with the results of Pagel and colleagues, where they also observed that nouns were most resistant to change, followed by verbs and adjectives [47].

**Figure 5:**
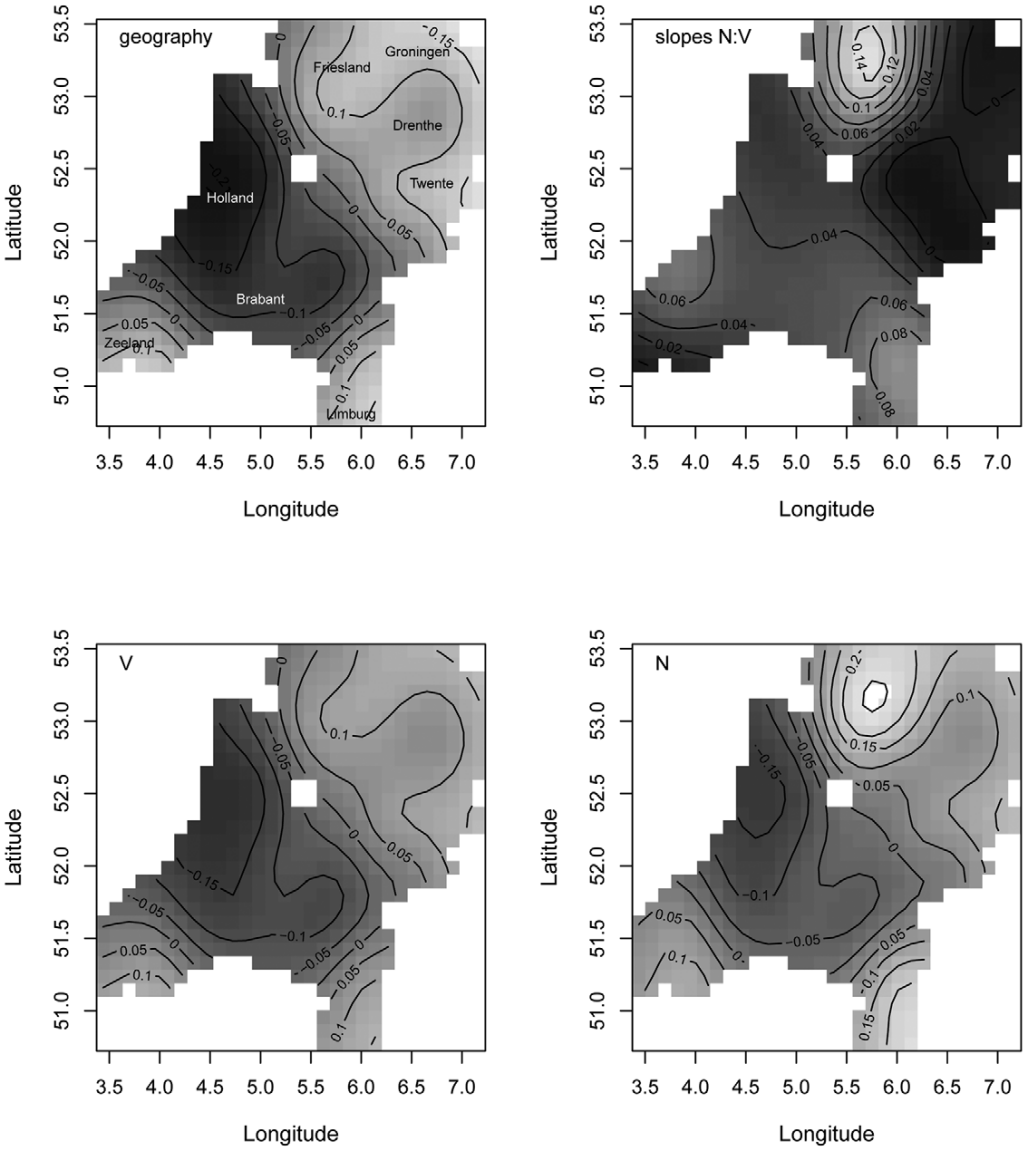
Contrast between nouns as opposed to verbs/adjectives and distance from the standard language. Upper left: distance predicted only from longitude and latitude. Upper right: the geographical distribution of random slopes for the contrast between nouns as opposed to verbs and adjectives. Bottom panels: the combined effect of geography and the word category on pronunciation distance for verbs/adjectives (panel V) and nouns (panel N). Darker shades of gray denote smaller values, lighter shades indicate larger values.

Similar to word frequency, we also observe a non-uniform effect of the contrast between nouns as opposed to verbs and adjectives across locations, indicated by the presence of the by-location random slopes for the word category contrast in our model (see Table 2). The parameters for these random slopes (the standard deviation for the random slopes and the correlation parameter for the random slopes and intercepts) jointly increase the log-likelihood of the model by 1064 units, compared to 5.6 log-likelihood units for the fixed-effect (population) slope of this contrast. These by-location random slopes are not uniformly distributed across the geographical area, as shown by the upper right panel of Figure 5. This panel clearly shows that the word category in the north-west of the Netherlands does not influence the distance from the standard language (i.e. the slope is 0), while in Friesland nouns have a much higher distance from the standard than verbs or adjectives.

To clarify how geography (GAM distance) and the word category contrast jointly predict distance from the standard language, we first calculated the fitted GAM distance for each location. We then estimated the predicted distance from the standard language using GAM distance, a fixed (median) word frequency, and the word category contrast as predictors, weighted by the weights estimated by our mixed-effects model. Because the fitted surfaces are different for nouns as opposed to verbs and adjectives, we visualized both surfaces in the bottom panels in Figure 5. The first thing to note is that in panel N the shades of grey are lighter than in panel V, indicating greater differences from the standard. This is the main effect of the word category contrast: nouns are more likely to resist assimilation to the standard language than verbs or adjectives. The second thing to note is that the distances between the contour lines are smaller for nouns, indicating that the differences between regions are more pronounced for nouns than for verbs.

As the pattern of variation at the periphery of the Netherlands is quite similar to the pattern reported for high-frequency words (i.e. the peripheral areas are quite distinct from the standard), we will not repeat its discussion here. The similarity between high-frequency words and nouns (as opposed to verbs and adjectives) is also indicated by the correlation parameter of 0.550 in Table 2.

## Discussion

In this study we have illustrated that several factors play a significant role in determining dialect distances from the standard language. Besides the importance of geography, we found clear support for three word-related variables (i.e. the contrast between nouns as opposed to verbs and adjectives, word frequency and the vowel-to-consonant ratio in the standard Dutch pronunciation) as well as two variables relating to the social environment (i.e. the number of inhabitants in a location and the average age of the inhabitants in a population). These results clearly indicate the need for variationists to consider explanatory quantitative models which incorporate geographical, social and word-related variables as independent variables.

We did not find support for the importance of speaker-related variables such as gender and age. As we only had a single pronunciation per location, we cannot exclude the possibility that these speaker-related variables do play an important role. It would be very informative to investigate dialect change in a data set with speakers of various ages in the same location, using the apparent time construct [54]. In addition, being able to compare male and female speakers in a single location would give us more insight into the effect of gender.

It is important to note that the contribution of the random-effects structure to the goodness of fit of the model tends to be one or two orders of magnitude larger than the contributions of the fixed-effect predictors, with GAM distance (geography) as sole exception. This indicates that the variation across speakers/locations and across words is huge compared to the magnitude of the effects of the socio-demographic and lexical predictors.

Our model also provides some insight into lexical diffusion. While we did not focus on individual sound changes, it is clear that the resistance to change at the word level is influenced by several word-related factors, as well as a number of socio-demographic factors of which the precise effect varies per word. Consequently, it is sensible to presume that a sound in one word will change more quickly than the same sound in another word (i.e. constituting a lexically gradual change). However, to make more precise statements about lexical diffusion as opposed to the lexically abrupt sound changes posited in the Neogrammarian hypothesis (e.g., see [55] for a discussion of both views), it is necessary to look at the level of the individual sound correspondences.

It would, therefore, be rewarding to develop a model to predict if an individual sound in a dialectal pronunciation is equal to or different from the corresponding sound in the standard Dutch pronunciation. As the Levenshtein distance is based on the alignments of sounds, these sound correspondences are already available. Using a logistic mixed-effects regression model would enable us to determine which factors predict the (dis)similarity of this sound compared to the sound in the standard Dutch pronunciation. Of course, this would also increase the computational effort, but since on average every word consists of about 4 to 5 sounds, this potential study should remain tractable.

In the present study, we connected a larger distance from standard Dutch with a greater resistance to change (i.e. standardization). While this might be true, it is also possible that words do not only change in the direction of the standard language. Ideally this should be investigated using pronunciations of identical words at different moments in time. For example, by comparing our data to the overlapping older pronunciations in the *Reeks Nederlandse Dialectatlassen* [56].

Instead of using standard Dutch as our reference point, we could also use proto-Germanic, following the approach of Heeringa and Joseph [57]. It would be rewarding to see if smaller distances from the proto-language correspond to larger distances from the standard language. Alternatively, we might study the dialectal landscape from another perspective, by selecting a dialectal variety as our reference point. For example, dialect distances could be calculated with respect to a specific Frisian or Limburgian dialect.

In summary, our quantitative sociolinguistic analysis has found support for lexical diffusion in Dutch dialects and has clearly illustrated that convergence towards standard Dutch is most likely in low-frequent words. Furthermore we have shown that mixed-effects regression modeling in combination with a generalized additive model representing geography is highly suitable for investigating dialect distances and its determinants.
